# Development of Machine-Learning Model to Predict COVID-19 Mortality: Application of Ensemble Model and Regarding Feature Impacts

**DOI:** 10.3390/diagnostics12061464

**Published:** 2022-06-14

**Authors:** Seung-Min Baik, Miae Lee, Kyung-Sook Hong, Dong-Jin Park

**Affiliations:** 1 Division of Critical Care Medicine, Department of Surgery, College of Medicine, Ewha Womans University, Seoul 07804, Korea; illaillailla@naver.com (S.-M.B.); hongks@ewha.ac.kr (K.-S.H.); 2 Department of Laboratory Medicine, College of Medicine, Ewha Womans University of Korea, Seoul 07804, Korea; miae@ewha.ac.kr

**Keywords:** COVID-19, mortality, artificial intelligence, ensemble model

## Abstract

This study was designed to develop machine-learning models to predict COVID-19 mortality and identify its key features based on clinical characteristics and laboratory tests. For this, deep-learning (DL) and machine-learning (ML) models were developed using receiver operating characteristic (ROC) area under the curve (AUC) and F1 score optimization of 87 parameters. Of the two, the DL model exhibited better performance (AUC 0.8721, accuracy 0.84, and F1 score 0.76). However, we also blended DL with ML, and the ensemble model performed the best (AUC 0.8811, accuracy 0.85, and F1 score 0.77). The DL model is generally unable to extract feature importance; however, we succeeded by using the Shapley Additive exPlanations method for each model. This study demonstrated both the applicability of DL and ML models for classifying COVID-19 mortality using hospital-structured data and that the ensemble model had the best predictive ability.

## 1. Introduction

In December 2019, the World Health Organization (WHO) first reported pneumonia of an unknown cause, which was subsequently linked to a novel coronavirus [1]. In February 2020, the WHO officially identified severe acute respiratory syndrome coronavirus 2 (SARS-CoV-2) as the causative agent of coronavirus disease 2019 (COVID-19), which spread rapidly, prompting the WHO to declare a worldwide pandemic in March 2020. Despite the passage of more than two years since the outbreak, the pandemic persists, notably in Korea, where the number of COVID-19-related deaths recently increased sharply [2], and several trials have been conducted in various fields, including treatment [3,4].

Most cases of COVID-19 are asymptomatic or only involve mild symptoms [5,6,7,8,9,10]. Nevertheless, the number of critically ill patients and fatalities continues to increase worldwide. In January 2022, the WHO reported that more than 370 million people had developed COVID-19, and more than 5.6 million had died [11]. The U.S. Centers for Disease Control and Prevention reported that, among all COVID-19 patients in the United States, 14% required hospitalization, 2% required intensive care, and 5% died [12]. High mortality rates were reported in areas with a high incidence of COVID-19 [13,14]. An important contributing factor is the inability of limited medical resources to manage the rapidly increasing number of patients, which results in the failure to diagnose COVID-19 in a timely manner, thereby contributing to increased mortality.

The Sequential Organ Failure Assessment, Acute Physiology and Chronic Health Evaluation II, and Simplified Acute Physiology scores are commonly used as scales for mortality prediction in critically ill patients [15,16,17]. However, they have limitations in the risk stratification necessary for mortality prediction [18]. Recently, analyses of mortality prediction via machine learning (ML) and deep learning (DL) have been conducted in clinical settings. Multiple studies using ML demonstrated the successful prediction of COVID-19 severity and mortality [19,20,21]. An ML study predicted the deterioration of a patient’s condition using demographic and comorbidity data and vital signs [22]. In addition, several DL models were published [23,24]. However, there have been few ML or DL studies of COVID-19 conducted in Korea.

ML facilitates the resolution of unknown patterns in data by learning training data and making models to achieve better predictions [25,26]. Several ML algorithms (e.g., support vector machine (SVM), random forest (RF), and boosting models) were used in biology, genomics, and toxicology [27,28,29,30,31]. In medical fields, with the exception of image and text data in medical charts, most laboratory tests use data from blood or urine samples, which are mainly composed of numerical inputs [32]. A deep neural network (DNN), a type of DL that uses hidden layers [33,34], is renowned for its ability to analyze high-dimensional data. Because bioinformatic data are generally high-dimensional, a DNN may be a suitable model for the study of bioinformatics [35,36].

There are several studies on the usefulness of DL using image data from COVID-19 patients [37,38]; however, studies using numerical data, which account for most medical data, are still insufficient.

This study was performed to analyze the electronic medical record data and laboratory findings of hospitalized COVID-19 patients in Korea using ML and DL to develop an optimized model for mortality prediction.

## 2. Materials and Methods

**Figure 1:**
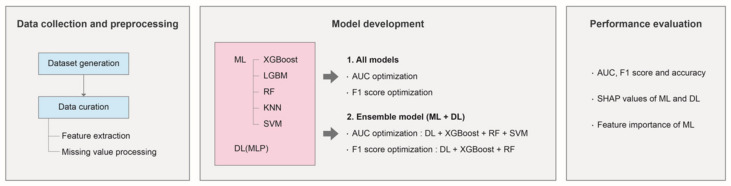
Schematic overall flow chart of conducting the study.

The principal procedures of the machine-learning prediction model for COVID-19 mortality are described in the following sections. The overall workflow is demonstrated in Figure 1.

### 2.1. Patients and Data Collection

This study included patients diagnosed with COVID-19 who were admitted to the sub-intensive and intensive care units between September 2021 and January 2022. All had moderate-to-severe COVID-19. Moderate severity was defined as requiring oxygen supplied by a high-flow system or mechanical ventilation. Data were collected regarding the following clinical characteristics: sex, age, medical history, vital signs, chief complaints, review of systems, and mortality.

Data were also collected regarding the following laboratory data: complete blood count with differential count, prothrombin time, activated partial thromboplastin time, total calcium, phosphorus, glucose, blood urea nitrogen (BUN), creatinine, estimated glomerular filtration rate, triglyceride, total cholesterol, total protein, albumin, aspartate aminotransferase (AST), alanine aminotransferase (ALT), alkaline phosphatase, total bilirubin, sodium, potassium, chloride, total carbon dioxide (CO_2_), magnesium, amylase, lipase, C-reactive protein, ammonia, arterial blood gas analysis, lactate, creatinine kinase (CK), CK-MB, high sensitivity troponin T, procalcitonin, lactate dehydrogenase, N-terminal pro-B-type natriuretic peptide, uric acid, serum osmolarity, ferritin, fibrinogen, fibrinogen degradation production, and D-dimer.

All clinical characteristics and laboratory findings were the initial values obtained after admission. This study was approved by the Institutional Review Board (IRB) of Ewha Womans University Mokdong Hospital (approval number: EUMC 2022-01-031), which waived the need for informed consent. All methods and datasets were carried out in accordance with relevant guidelines and regulations by the IRB.

### 2.2. Statistical Data Analyses

COVID-19 mortality was evaluated using the chi-squared test and the Student’s *t*-test. All statistical analyses were performed using IBM SPSS software, version 26.0 (SPSS Inc., Chicago, IL, USA).

### 2.3. DL Model Selection and Training

The DL model was shown to approximate the function of a complex structure [39,40,41]. Ours was conducted using a multi-layer perceptron (MLP), a class of DNN, for classification [42,43]. An MLP consists of at least three layers: input, hidden, and output. In this study, the hidden layer comprised one layer to which we applied the dropout technique, a simple method to prevent overfitting in neural networks [44,45,46]. Our MLP model was updated using the Adam optimizer, a method of stochastic optimization that uses a calculated parameter gradient.

### 2.4. ML Model Selection

Several ML models were used for disease prediction. Among the decision-tree models, the bagging and boosting ML models demonstrated a robust classification of COVID-19 mortality. In addition, SVM and K-nearest neighbors (KNN) models were used to create an optimized model for mortality classification. In this study, there were 8.0% missing values in 17,052 datasets from 203 COVID-19 cases.

### 2.5. ML Model Development

Tree-based models can be implemented regardless of missing values. However, SVM and KNN models cannot; instead, the missing values are replaced with the median values of each test result. Here, we used the following ML models: RF, Extreme Gradient Boosting (XGBoost), Light Gradient Boosting Machine (LGBM), SVM, and KNN.

### 2.6. Development of an Ensemble Model Using DL and ML

An ensemble model was developed with the best-performing ML models and a DL (MLP) model to achieve optimized results. First, the area under the receiver operating characteristic curve (AUC) optimization was conducted by integrating the results of four models: three ML models (XGBoost, RF, and SVM) and one DL model (MLP). Second, for F1 score optimization, an ensemble model was developed by hard voting that involved the top-three F1 score models: XGBoost, RF, and DL. The ensemble model was developed by using only the results identified as COVID-19 mortality in ≥2 models to determine the optimized F1 score.

### 2.7. Performance Measurement

We used the F1 score, accuracy, and AUC data as performance measures for the model, which was evaluated using the AUC analysis provided by Python Library.

### 2.8. Shapley Additive exPlanations (SHAP) Method

The SHAP method is a new technique for estimating the influence of each feature according to a probabilistic game rule [47,48]. In DL models, it is impossible to determine the extent to which each parameter (feature) contributes to completed model performance because DL models are effectively “black box” systems. In order to solve this problem, we used the SHAP method, which was also used to predict the feature impacts of three ML models: XGBoost, LGBM, and RF.

### 2.9. Feature Extraction

Feature extraction is important for developing and measuring the performances of ML models, and in our study, we identified three new parameter ratios: pulse rate (PR)/respiratory rate (RR), AST/ALT, and neutrophil/lymphocyte.

### 2.10. Standard Scaling

As a data pre-processing step during ML and DL model training, the range of columns in the COVID-19 dataset was standardized and scaled using “scikit-learn” in the Python Library.

### 2.11. Cross-Validation

For K-fold cross-validation, we divided the total dataset of 203 COVID-19 patients to create the training and validation sets using a ratio of 4:1, respectively. Because we had a limited number of cases, the test set itself may not have fully represented all possible clinical characteristics and laboratory findings. Thus, K-fold validation (n_split:5) was applied to the entire dataset to overcome this limitation.

## 3. Results

### 3.1. Demographic and Clinical Characteristics

This study included 203 Korean patients between September 2021 and January 2022 who had moderate-to-severe COVID-19. Of these, 49 (23.1%) were in the non-survivor group, and 154 were in the survivor group (76.9%). The mean age was significantly older in the non-survivor group (*p* < 0.05). The initial demographics and clinical characteristics of the two groups are shown in Table 1.

### 3.2. Data Features and Parameters

For ML model evaluation, because of the data imbalance between the non-survivor and survivor groups, we evaluated the performances of individual models using the AUC and F1 scores.

In our study, 84 data parameters were collected for each patient: 65 laboratory test results and 19 that included medical history obtained from the patients’ electronic medical records, blood pressure, body temperature, and arterial blood gas analysis (Table 1, Supplementary Table S1). Three new parameters were obtained by feature extraction: the AST/ALT, neutrophil/lymphocyte, and PR/RR ratios, which were used to develop the ML models, including the MLP of the DL model. Thus, 87 mostly numerical and structured parameters were used for model development, with the goal of creating a classifier ML model and a neural network (i.e., a DL model) to distinguish between non-survivor and survivor groups.

### 3.3. DL (Neural Network) Performance Optimized by the AUC and F1 Score

**Figure 2:**
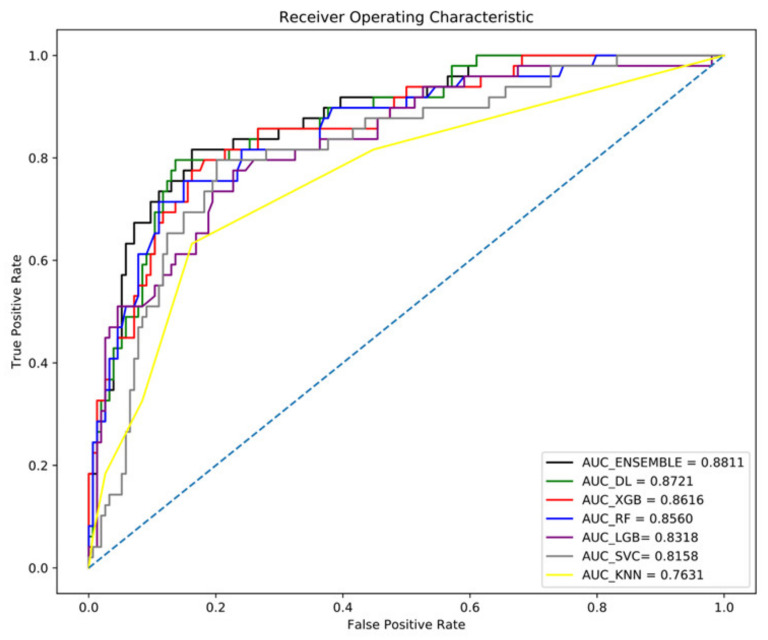
Area under the receiver operating characteristic curves (AUC) of machine-learning models, deep-learning model, and an ensemble model.

We developed a new DL neural network and trained it using the 87 parameters. AUC optimization revealed a value of 0.8721, an accuracy of 0.84, an F1 score of 0.76, a precision of 0.79, and a recall of 0.74 (Figure 2 and Table 2). The DL model exhibited the best accuracy, F1 score, and recall compared to the ML models; thus, DL performed better than ML for classifying numerical and structured patient data.

In addition, optimized cut-offs were determined for each ML model, and hyperparameter tuning was used to achieve an optimized F1 score. The optimized DL model had an F1 score of 0.78, an AUC of 0.8614, an accuracy of 0.83, a precision of 0.77, and a recall of 0.79 (Table 3). The F1 score for the DL optimization model was 0.78, which was the same as RF, the highest result (Table 3). The accuracy of our DL model was 0.83, and its results were similar to the findings of the tree-based models (RF, XGBoost, and LGBM).

### 3.4. Performances of ML Models Optimized by AUC

Tree-series models generally demonstrate good performance in solving classification problems [49,50,51]. Therefore, we developed ML models that used tree-based models (RF, XGBoost, and LGBM) and other ML approaches (SVM and KNN). The performance results for each model are described in Table 2. The best performance among the ML models was demonstrated by XGBoost (AUC: 0.8616), a boosting series of tree-based models; the next best performances were by RF (AUC: 0.8560), the boosting model LGBM (AUC: 0.8318), SVM (AUC: 0.8158, based on the relative distance between data), and the KNN model (AUC: 0.7631) (Figure 2). The RF and LGBM models demonstrated the highest accuracy (0.83), while XGBoost (0.82), SVM (0.81), and KNN (0.79) were slightly lower (Table 2).

### 3.5. ML Model Results Optimized According to F1 Score

In this study, data imbalance was present between the non-survivor and survivor groups. Thus, the F1 score was used as the evaluation index for model performance evaluation and optimization (Table 3). Similar to the AUC results, the DL model showed the best F1 score. Among the ML models, RF had the highest F1 score (0.78) followed by XGBoost (0.77), SVM (0.76), LGBM (0.75), and KNN (0.72) (Table 3).

### 3.6. Ensemble Model of DL and ML Models

Because DL exhibited good performance in AUC analyses, we developed a new ensemble model that blended the DL model with three ML models based on their AUC outcomes. The greatest weight was placed on the DL model, followed by XGBoost, RF, and SVM. The highest-performing ensemble model achieved the best AUC result (0.8811) and demonstrated the highest accuracy (0.85) (Figure 2 and Table 2). Finally, we created an optimal AUC ensemble model that showed 85% accuracy, 81% precision, 75% recall, and an F1 score of 77% (Table 2).

Additionally, to achieve F1 score optimization through the ensemble, we selected three models—RF, XGBoost, and the DL model with the highest F1 score. We used the hard voting technique to vote in two or more models. The resulting highest F1 score was 0.80 (Table 3). The DL model showed a better AUC and F1 score than the ML model. As a result, it had better performance (Figure 2 and Table 2 and Table 3).

### 3.7. Feature Importances in ML Models

**Figure 3:**
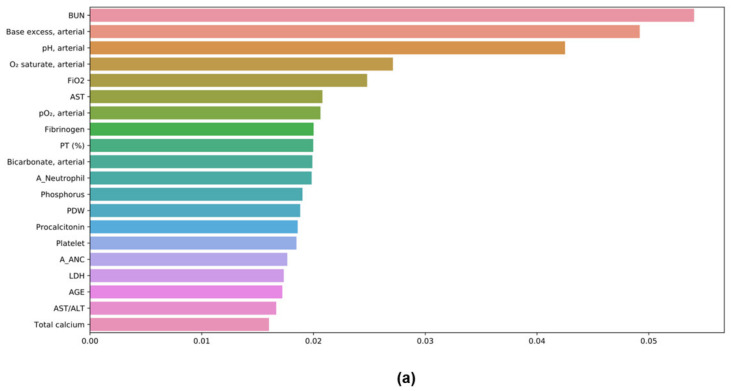

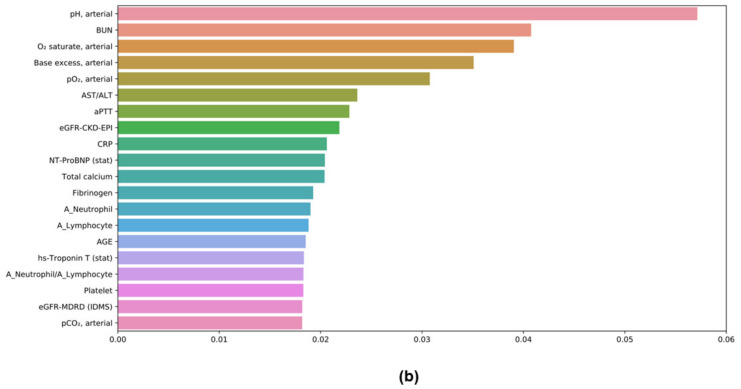
Feature importance of XGBoost (**a**) and RF (**b**) in COVID-19 mortality.

The top 20 important features in the RF and XGBoost models are shown in Figure 3. In XGBoost, BUN was the most important; the others were base excess, pH, saturation of percutaneous oxygen (SpO_2_), partial pressure of oxygen (PO_2_), and procalcitonin. In the RF model, pH was the most important feature, followed by BUN, SpO_2_, base excess, PO_2_, and total calcium.

### 3.8. Evaluation of the Model Performance Impact When Using the Mean SHAP Value in DL and ML Models

**Figure 4:**
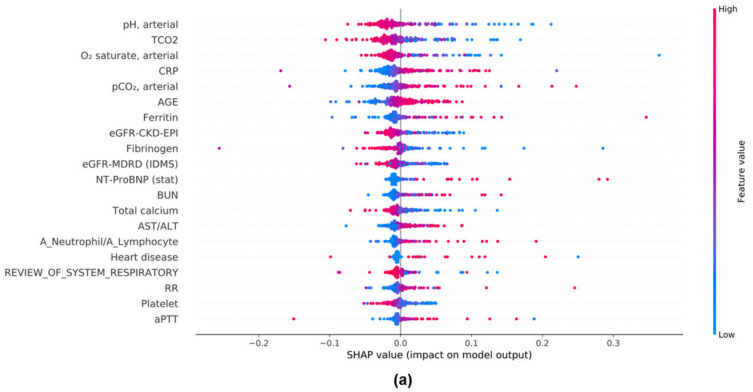

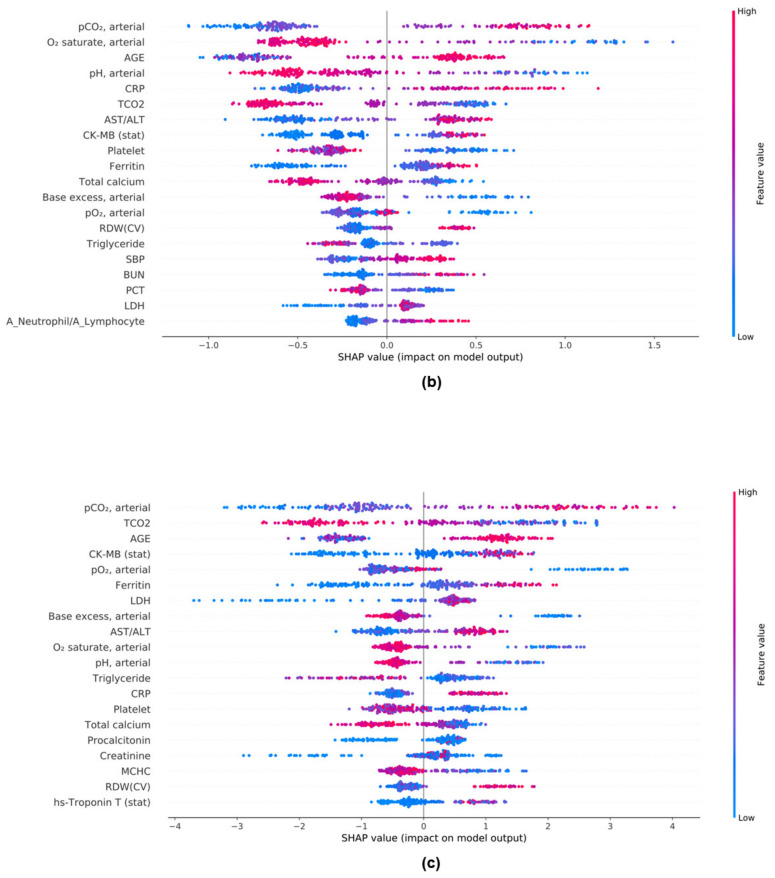

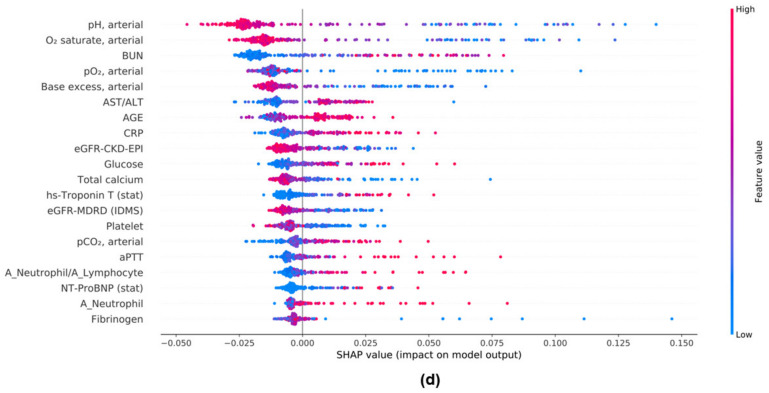
Analysis of features contributing to COVID-19 mortality by the SHAP method. (**a**) DL model. (**b**) XGBoost model. (**c**) LGBM model. (**d**) RF model.

The SHAP method provided the means for assessing the effects of features on classification targets. We analyzed the mean SHAP values of the DL and three ML models to identify features associated with COVID-19 mortality. The top 20 features that affected each model are shown in Figure 4. In DL and RF, pH was the most important feature; it was negatively associated with disease outcomes in the non-survivor group. In LGBM and XGBoost, partial pressure of carbon dioxide (PCO_2_) was the most important feature and was positively associated with disease outcomes in the non-survivor group. The newly identified variable, the AST/ALT ratio, was included in the top 20 features of DL, RF, XGBoost, and LGBM models.

## 4. Discussion

In this study, we used 87 parameters, including laboratory tests and clinical characteristics, to develop an ensemble model from DL and ML models to predict COVID-19 mortality. Although the study did not include a large number of patients, a large and varied parameter set was used to develop predictive artificial intelligence models; additionally, the model was trained by performing K-fold validation to avoid loss in the test set. In this study, a DL model and several ML models were evaluated using AUC analysis and the F1 score. The DL model showed the best performance for accuracy and F1 score compared to the ML models (XGBoost, LGBM, RF, SVM, and KNN, Table 2 and Table 3). 

Several studies used ML or DL alone to predict COVID-19 mortality, such as an ML study by Vaid A et al., to predict mortality in 4098 patients [52]. According to reports, XGBoost was the best, with an AUC of 0.84 to 0.90, which was similar to our results (Figure 2). Li, Xiaoran et al., reported a mortality prediction model using deep learning for 1108 patients, of whom 142 died [53]. The AUC of the deep-learning model in this study was 0.844, which was lower than that of our DL and ensemble models (Figure 2). Although our study was conducted with a relatively small number of cases, the model performances were similar to those of other studies. There are two possible reasons.

First, the tree-series models generally demonstrated good performance with categorical structured data. However, the DL model performed better than the ML models because the data from our study consisted mostly of laboratory test results, which comprise numerical data. Categorical parameters, including sex and medical history (e.g., diabetes, hypertension, and heart disease), were converted into binary data using a label-encoding library. Therefore, compared to DL, the potential gain from the ML tree models was greatly reduced. Specifically, the shallow MLP of the DL model was effective for solving the classification problem because most components of hospital data are numerical. This result is consistent with the findings of other studies that used numerical hospital data [32].

The second reason is that the number of columns (parameters) was comparatively higher than the number of rows (number of patient cases) in our data structure. When there are comparatively more parameters than cases, a tree model is more likely to demonstrate poor performance because of overfitting. Because the number of cases was small and the number of columns large, there was an increasing number of questions during tree creation, so the tree became deeper. Therefore, dimensionality may impede model learning or cause overfitting. The DL model of the MLP was a combination of multiple layers of linear models. Thus, the number of columns was much less important in the linear model than in the tree model. Accordingly, the MLP showed good performance. However, the best performance was obtained when one hidden layer of the MLP was used. When two or more hidden layers were stacked, the performance was poor because of overfitting.

The dropout technique was notably used in this study. Without it, our model would have had to learn mainly through the features (512 nodes) that are considered important in the training set. This can lead to overfitting, resulting in poor outcomes in the test set. The advantage of the dropout method is that the model learns from fewer features (nodes) that are considered important but also from features (nodes) that are presumably negligible; thus, it has generalized power to provide a good fit even if unusual data subsequently enter the test set.

We developed ensemble machine-learning models to predict COVID-19 mortality using a DL model combined with several ML models. We found that the ensemble model of DL and ML achieved the optimal classification results in hospital-structured data (Table 2 and Table 3).

DL models generally cannot extract feature importance because they use a “black-box” approach; however, we did so through the SHAP method for each model, including the DL model. For data analysis and interpretation, the SHAP method was better than the widely used feature importance technique because it only lists the feature importance order for each model; it cannot accurately visualize whether each feature has a positive or negative influence on the classification target. In contrast, the SHAP method can indicate the degree and direction of each parameter’s effects on the classification target. In the DL model with the best performance, pH had the highest impact; in the non-survivor group, pH demonstrated a negative association with disease outcomes because of its relationship with acidosis, which is strongly associated with patient mortality [54]. Total CO_2_ also had a large impact. Because CO_2_ exists in the body as bicarbonate, analyzing total CO_2_ provides information on the patient’s acid–base balance; a lower total CO_2_ value is associated with a greater likelihood of acidosis [55]. Total CO_2_ also demonstrated a negative association in the non-survivor group, which suggested acidosis. Although the Student’s *t*-test found that the PCO_2_ level did not differ significantly between the two groups, SHAP revealed a positive association and high impact in the non-survivor group. High PCO_2_ levels are associated with respiratory acidosis and may lead to mortality. This study was conducted by generating new variables from existing laboratory findings. Among them, the neutrophil/lymphocyte ratio and the AST/ALT ratio showed a positive association with disease outcomes in the non-survivor group.

As is the case with other COVID-19 patients [56], a decrease in lymphocytes and an increase in neutrophils were observed in our study, but the levels between the two groups differed significantly; however, these variables demonstrated no feature effect in the DL model, as determined using the SHAP method. However, the neutrophil/lymphocyte ratio had a large impact. In addition, feature analysis in XGBoost, LGBM, and RF models was performed using the SHAP method. In the RF, XGBoost, and DL models, BUN had a high impact and showed a positive association with disease outcomes in the non-survivor group (Figure 4a,b,d), but high BUN levels also indicated decreased kidney function. COVID-19 causes sepsis, which causes dysfunction in various organs, including the kidneys. In patients with sepsis, fluid demand may increase [57], and BUN rises in the absence of an adequate fluid supply. Indeed, BUN was significantly higher in the non-survivor group. In the XGBoost, LGBM, RF, and DL models, the AST/ALT ratio showed a positive association with disease outcomes in the non-survivor group. Elevated AST and ALT were also observed [56], and in our study, these levels tended to be higher in the non-survivor group, but the results were not statistically significant. However, the AST/ALT ratio was significantly higher in the non-survivor group; its feature impact was high in the XGBoost, LGBM, RF, and DL models. The importance of AST and ALT in respiratory diseases may be low in clinical practice; however, we found that they were important indicators for COVID-19 mortality. In contrast to tree-based ML models (e.g., XGBoost, LGBM, and RF), only the DL model demonstrated how features affect heart disease and a patient’s general condition. Our findings confirmed that capabilities differed between DL and ML models.

In summary, this study demonstrated the application of DL (MLP) and ML in classifying COVID-19 mortality using numerical data (e.g., laboratory test results and clinical characteristics) from hospitalized patients. Although the performances of the ML models were relatively good, the DL model performed the best overall. In addition, using the DL and ML ensemble model, we achieved the most optimized performance. Finally, our approach identified several novel parameters for predicting COVID-19 mortality.

In conclusion, we demonstrated that DL and ML obtain favorable outcomes for mortality prediction using hospital data. Our ML models shed new light on the values of clinical information and laboratory tests that had not been previously highlighted.

## Figures and Tables

**Table 1:** Demographics and clinical characteristics of COVID-19 patients.

Variable	Total (*n* = 203)	Non-Survival (*n* = 49)	Survival (*n* = 154)	*p*
Male sex (%)	98 (48.3)	24 (49.0)	74 (48.1)	0.910
Age (years)	67.5 ± 16.16	75.7 ± 11.58	64.9 ± 16.57	<0.05
Hospitalization period	13.4 ± 10.20	16.8 ± 12.81	12.4 ± 9.00	<0.05
Comorbidities (%)				
Hypertension	98 (48.3)	30 (61.2)	68 (44.2)	0.055
Diabetes mellitus	56 (27.6)	20 (40.8)	36 (23.4)	<0.05
Heart disease	6 (3.0)	6 (12.2)	10 (6.5)	0.319
Lung diasease	12 (5.9)	3 (6.1)	9 (5.8)	0.943
Liver disease	5 (2.5)	0 (0)	5 (3.2)	0.454
Kidney disease	5 (2.5)	2 (4.1)	3 (1.9)	0.756
Brain disease	27 (13.3)	6 (12.2)	21 (13.6)	0.993
Malignant disease	28 (13.8)	7 (14.3)	21 (13.6)	0.909
Vital signs at hospital admission				
Systolic blood pressure (mmHg)	133.4 ± 22.08	132.9 ± 25.12	133.6 ± 21.10	0.866
Diastolic blood pressure (mmHg)	79.3 ± 14.76	75.2 ± 14.22	80.6 ± 14.74	<0.05
Pulse rate (PR, bpm)	84.4 ± 17.79	84.3 ± 23.84	84.5 ± 15.53	0.962
Respiratory rate (RR, bpm)	22.4 ± 10.03	25.6 ± 17.10	21.3 ± 6.17	0.095
Body temperature (°C)	37.1 ± 0.71	36.9 ± 0.82	37.1 ± 0.65	<0.05
Pulse pressure (mmHg)	54.1± 17.88	57.7 ± 20.23	53.0 ± 16.99	0.147
PR/RR	4.1 ± 1.34	3.9 ± 1.74	4.2 ± 1.18	0.316

**Table 2:** DL and ML performances by AUC optimization.

Classifier	AUC	Accuracy	F1-Score	Precision	Recall
XGboost	0.8616	0.82	0.75	0.8	0.73
LGBM	0.8318	0.83	0.71	0.83	0.68
RF	0.8560	0.83	0.74	0.80	0.71
KNN	0.7631	0.79	0.59	0.77	0.58
SVM	0.8158	0.81	0.72	0.74	0.71
DL	0.8721	0.84	0.76	0.79	0.74
Ensemble model *	0.8811	0.85	0.77	0.81	0.75

DL: deep learning; ML: machine learning; XGboost: extreme gradient boosting; LGBM: light gradient boosting model; RF: random forest; KNN: K-nearest neighbors; SVM: support vector machine. * Ensemble model of deep-learning and machine-learning models.

**Table 3:** DL and ML performances by F1 score optimization.

Classifier	AUC	Accuracy	F1-Score	Precision	Recall
XGboost	0.8331	0.83	0.77	0.76	0.77
LGBM	0.8318	0.85	0.75	0.84	0.72
RF	0.8560	0.84	0.78	0.78	0.77
KNN	0.7631	0.79	0.72	0.72	0.74
SVM	0.8158	0.82	0.76	0.76	0.76
DL	0.8614	0.83	0.78	0.77	0.79
Ensemble model *	0.8631	0.85	0.80	0.80	0.80

DL: deep learning; ML: machine learning; XGboost: extreme gradient boosting; LGBM: light gradient boosting model; RF: random forest; KNN: K-nearest neighbors; SVM: support vector machine. * Ensemble model of deep-learning and machine-learning models.

## Data Availability

Data are available upon reasonable request.

## Supplementary Materials

The following supporting information can be downloaded at: https://www.mdpi.com/article/10.3390/diagnostics12061464/s1, Table S1: Laboratory results of COVID-19 patients.
