# Supplementary material for: Development of Machine-Learning Model to Predict COVID-19 Mortality: Application of Ensemble Model and Regarding Feature Impacts
Source: Diagnostics (Basel). 2022 Jun 14;12(6):1464. doi: 10.3390/diagnostics12061464 (PMC9221552; doi:10.3390/diagnostics12061464)
Supplement: Supplementary file 1 [file diagnostics-12-01464-s001.zip › diagnostics-1770754-supplementary.pdf]

Table S1. Laboratory results of COVID-19 patients.

Variable	Total (n = 203)	Non-survival (n = 49)	Survival (n =154)	*p*
White blood cell count ($\times10^3$/uL)	8.59 ± 5.059	10.23 ± 6.992	8.06 ± 4.148	< 0.05
Red blood cell count ($\times10^6$/uL)	4.03 ± 0.719	3.82 ± 0.714	4.10 ± 0.709	< 0.05
Hemoglobin (g/dL)	12.10 ±2.144	11.44 ± 2.073	12.32 ± 2.129	< 0.05
Hematocrit (%)	35.1 ± 5.77	33.4 ± 5.66	35.6 ± 5.73	< 0.05
Mean corpuscular volume (fL)	87.5 ± 5.67	88.1 ± 6.22	87.3 ± 5.49	0.417
Mean corpuscular hemoglobin (pg)	30.1 ± 2.26	30.1 ± 2.43	30.1 ± 2.21	0.942
Mean corpuscular hemoglobin concentration (%)	34.4 ± 1.35	34.2 ± 1.35	34.5 ± 1.35	0.133
Red cell distribution width	13.3 ± 1.66	13.8 ± 1.83	13.2 ± 1.58	< 0.05
Platelet ($\times10^3$/uL)	211.2 ± 96.92	165.1 ± 80.58	226.1 ± 97.28	< 0.05
Plateletcrit (%)	0.22 ± 0.092	0.18 ± 0.079	0.23 ± 0.093	< 0.05
Mean Platelet Volume (fL)	10.4 ± 1.00	10.7 ± 0.89	10.2 ± 1.01	< 0.05
Platelet volume distribution width (fL)	11.7 ± 2.49	12.4 ± 2.49	11.5 ± 2.46	< 0.05
Automated absolute neutrophil count ($\times10^3$/uL)	7.37 ± 4.879	9.17 ± 6.753	6.79 ± 3.954	< 0.05
Automated neutrophil (%)	82.7 ± 10.84	87.0 ± 8.53	81.3 ± 11.15	< 0.05
Automated lymphocyte (%)	11.5 ± 7.91	8.5 ± 6.87	12.4 ± 8.00	< 0.05
Neutrophil/lymphocyte	13.36 ± 17.687	23.30 ± 31.394	10.16 ± 7.661	< 0.05
Automated monocyte (%)	5.3 ± 3.68	4.2 ± 2.90	5.7 ± 3.84	< 0.05
Automated eosinophil (%)	0.3 ± 1.40	0.1 ± 0.62	0.4 ± 1.57	0.228
Automated basophil (%)	0.2 ± 0.22	0.2 ± 0.30	0.2 ± 0.19	0.291

Manual absolute neutrophil count (%)	6.9 ± 5.60	8.1 ± 7.05	5.9 ± 4.12	0.273
Manual neutrophil (%)	81.0 ± 12.84	86.6 ± 8.35	76.8 ± 14.15	< 0.05
Manual lymphocyte (%)	11.8 ± 7.55	7.8 ± 5.44	14.7 ± 7.63	< 0.05
Manual monocyte (%)	5.0 ± 4.23	3.5 ± 1.67	6.2 ± 5.24	< 0.05
Manual eosinophil (%)	1.50 ±.707	1.00	2.00	
Manual myelocyte (%)	3.9 ± 5.08	3.0 ± 3.37	4.8 ± 6.85	0.663
Activated Partial Thromboplastin Time	32.08 ± 10.791	35.98 ± 10.466	30.82 ± 10.632	< 0.05
Prothrombin Time (sec)	13.5 ± 2.51	14.5 ± 2.69	13.2 ± 2.38	< 0.05
Prothrombin Time (%)	83.2 ± 17.88	75.1 ± 22.28	85.7 ± 15.58	< 0.05
Prothrombin Time (INR)	1.146 ± 0.2262	1.231 ± 0.2351	1.120 ± 0.2178	< 0.05
Total calcium (mg/dL)	8.11 ± 0.620	7.74 ± 0.770	8.23 ± 0.513	< 0.05
Phosphorus (mg/dL)	3.09 ± 1.013	3.47 ± 1.277	2.97 ± 0.883	< 0.05
Glucose (mg/dL)	177.7 ± 78.28	209.9 ± 81.68	167.4 ± 74.52	< 0.05
Blood urea nitrogen (mg/dL)	23.2 ± 19.85	35.8 ± 22.67	19.2 ± 17.04	< 0.05
Creatinine (mg/dL)	0.955 ± 0. 6616	1.357 ± 0.9500	0.826 ± 0.4729	< 0.05
eGFR-MDRD -IDMS (mL/min/1.73 m2)	92.3 ± 45.55	65.5 ± 36.28	100.9 ± 44.96	< 0.05
eGFR-CKD-EPI (mL/min/1.73 m2)	81.8 ± 29.86	60.5 ± 30.10	88.7 ± 26.42	< 0.05
Triglyceride (mg/dL)	125.4 ± 73.17	130.4 ± 80.32	123.9 ± 71.25	0.630
Total cholesterol (mg/dL)	132.3 ± 40.64	115.0 ± 43.76	137.1 ± 38.52	< 0.05

Total protein (g/dL)	6.05 ± 0.693	5.71 ± 0.800	6.16 ± 0.619	< 0.05
Albumin (g/dL)	3.28 ± 0.515	3.02 ± 0.473	3.37 ± 0.500	< 0.05
Aspartate aminotransferase (IU/L)	65.9 ± 155.15	113.4 ± 306.37	50.7 ± 35.10	0.160
Alanine aminotransferase (IU/L)	42.5 ± 59.97	48.7 ±100.11	40.4 ± 39.68	0.574
AST/ALT	1.78 ± 1.061	2.35 ± 1.272	1.60 ± 0.917	< 0.05
Alkaline phosphatase (IU/L)	91.0 ± 53.32	100.9 ± 69.18	87.8 ± 46.93	0.222
Total bilirubin (mg/dL)	0.49 ± 0.365	0.58 ± 0.542	0.47 ± 0.283	0.151
Sodium (mmol/L)	136.7 ± 4.70	137.4 ± 5.53	136.5 ± 4.40	0.241
Potassium (mmol/L)	4.21 ± 0.686	4.51 ± 0.832	4.12 ± 0.605	< 0.05
Chloride (mmol/L)	103.9 ± 5.31	105.4 ± 5.00	103.4 ± 5.34	< 0.05
Total carbon dioxide (mmol/L)	19.0 ± 3.55	17.0 ± 3.52	19.7 ± 3.30	< 0.05
Amylase (IU/L)	95.7 ± 90.43	106.5 ±122.03	81.3 ± 40.61	0.750
Lipase (U/L)	151.9 ± 250.31	190.8 ± 334.00	100.0 ± 116.48	0.677
C-reactive protein (mg/dL)	9.98 ± 7.396	14.40 ± 7.623	8.51 ± 6.730	< 0.05
Ammonia (umol/L)	39.700 ±22.7581	43.750± 30.6177	31.600±	
Creatine kinase (IU/L)	339.1 ± 1156.16	461.1 ± 674.48	303.2 ± 1263.82	0.492
Creatine kinase-MB (ng/mL)	3.19 ± 7.245	4.59 ± 4.002	2.78 ± 7.920	0.209
High sensitivity-Troponin T (ng/mL)	0.040 ± 0.1756	0.130 ± 0.3598	0.014 ± 0.0173	0.058
Procalcitonin (ng/mL)	2.358 ± 8.8979	8.365 ± 16.776	0.972 ± 4.9181	< 0.05
lactate dehydrogenase (IU/L)	455.5 ± 222.09	605.5 ± 314.37	418.3 ± 175.36	< 0.05

N-terminal pro-B-type natriuretic peptide (pg/mL)	1774 ± 4425.3	5086 ± 7426.8	825 ± 2380.8	< 0.05
Uric acid (mg/dL)	4.08 ± 2.256	5.05 ± 2.501	3.80 ± 2.112	< 0.05
Osmolality, serum (mOsm/kg)	291.9 ± 31.13	310.0 ± 29.51	282.8 ± 30.11	0.240
Ferritin (ng/mL)	1281 ± 1320.7	2011 ± 2039.8	1085 ± 970.5	< 0.05
Fibrinogen (mg/dL)	452.58 ± 156.614	441.14 ± 187.846	456.19 ± 146.144	0.167
Fibrinogen degradation production (ug/mL)	10.6 ± 18.47	19.4 ± 27.03	8.2 ± 14.43	< 0.05
D-dimer (mg/L)	4.31 ± 8.691	7.43 ± 11.492	3.49 ± 7.652	0.074
**Arterial blood gas analysis**				
pH	7.439 ± 0.0763	7.406 ± 0.0952	7.450 ± 0.0656	< 0.05
pCO_2 (mmHg)	33.62 ± 6.935	35.43 ± 9.552	33.01 ± 5.724	0.099
pO_2 (mmHg)	95.37 ± 38.929	92.04 ± 37.709	96.47 ± 39.387	0.496
Bicarbonate (mmol/L)	22.30 ± 3.671	21.66 ± 3.730	22.51 ± 3.639	0.162
Base excess (mmol/L)	-1.38 ± 4.163	-2.63 ± 4.362	-0.96 ± 4.024	< 0.05
CO_2 content (mmol/L)	23.32 ± 3.772	22.75 ± 3.840	23.52 ± 3.743	0.220
O_2 saturation (%)	95.34 ± 5.825	93.42 ± 9.361	95.99 ± 3.855	0.068
Lactate (mg/dL)	17.16 ± 16.639	19.96 ± 28.846	16.11 ± 8.371	0.225
